# The Treatment of Primary Biliary Cholangitis: Time for Personalized Medicine

**DOI:** 10.1007/s12016-025-09074-x

**Published:** 2025-07-12

**Authors:** Xinyi Men, Yansheng Liu, Han Zhao, Bingrui Xie, Changcun Guo, Patrick S. C. Leung, Suraj Timilsina, M. Eric Gershwin, Yulong Shang, Ying Han

**Affiliations:** 1 National Clinical Research Center for Digestive Diseases and Xijing Hospital of Digestive Diseases, Xijing Hospital, Air Force Medical University, Xi’an, China; 2 Division of Rheumatology, Allergy and Clinical Immunology, University of California, Davis, Davis, CA USA

**Keywords:** Primary Biliary Cholangitis (PBC), Incomplete response to UDCA, Refractory PBC, Early identification, Risk stratification, Intensive treatment, Precise treatment

## Abstract

Primary Biliary Cholangitis is an autoimmune liver disease distinguished by Anti-mitochondrial Antibodies and chronic non-suppurative lymphocytic microcholangitis. UDCA remains the exclusively recommended initial therapy for PBC. However, 40% of patients experience either incomplete biochemical response or intolerance to UDCA, which represents poorer outcomes and increased mortality. Therefore, early identification of high-risk patients and timely intensive treatments are necessary to delay the progression of PBC and better improve the prognosis. Intensive therapeutic strategies based on more stringent treatment goals and early efficacy assessment criteria are elaborated in this review. To exclude AIH-PBC overlap syndrome, liver biopsy is required for cholestasis patients with negative AMAs or PBC patients who respond inadequately to UDCA, especially those with elevated ALT and IgG. Combined immunosuppressants are considered for patients with moderate-to-severe hepatitis. ALP normalization is considered the improved therapeutic goal for high-risk patients, which has been verified achievable in multiple treatment attempts. The control of pruritus and fatigue constitutes the key therapeutic targets in the symptom management of PBC. Bezafibrate, Seladelpar, and IBAT inhibitors have demonstrated significant therapeutic potential in pruritus. Last but not least, Liver Stiffness Measurement is substantiated as efficient in the fibrotic monitoring of PBC patients. OCA and fibrates are respectively useful for compensated and decompensated fibrotic patients. Moreover, the conventional efficacy assessment procedure, which is the “wait-to-fail” strategy, exhibits suboptimal sensitivity in the timely detection of treatment-responsive patients. Therefore, early prediction and evaluation criteria at baseline and 1-month treatment will help in timely interventions for patients with insufficient efficacy. This improved identification strategy is expected to provide precise and personalized treatment for PBC patients.

## Introduction

Primary Biliary Cholangitis (PBC) is an autoimmune liver disease distinguished by high titer positive Anti-mitochondrial Antibodies (AMAs) and specific antinuclear antibodies (anti-sp100, anti-gp210), small intrahepatic bile duct injury, cholestasis, progressive liver fibrosis, and eventually decompensated liver cirrhosis [1]. The estimated incidence of PBC ranges from 0.84 to 2.75 per 100,000 due to geographical and ethnic differences [2], while the mortality rate is about three times higher than that of the general public [3]. Women account for 80 to 90% of patients with PBC [4], while the median/mean onset age ranges from 54 to 64 years [5]. The most prevalent clinical feature of PBC is fatigue and secondly pruritus, which manifests in approximately 70% of the patients [6]. Others include jaundice, thirst, hyperpigmentation, xerophthalmia, xanthelasmas, bone pain, and right upper abdominal discomfort [7]. 33.5% ~ 76% of PBC patients may present with hypercholesterolemia [8, 9]; however, most studies indicate that PBC patients exhibit no significant elevation in cardiovascular event risks (except for those with concomitant metabolic syndrome, who exhibit elevated risks), which is potentially attributable to the distinctive lipid profile of PBC patients [10, 11]. Conversely, certain investigations have reported a positive correlation between PBC and an increased cardiovascular risk [12, 13], suggesting the presence of complex, yet-undefined pathophysiological mechanisms. Studies have indicated an association between Systemic Rheumatic Diseases (SRDs) and PBC. Mendelian randomization analysis demonstrated a causal association between PBC and SRDs, with PBC exhibiting pathogenic effects on SRD development [14, 15], among which Sjogren’s syndrome is the most prevalent, with a pooled prevalence of 35% [16]. Without effective therapy and appropriate management, the progression to end-stage liver diseases—including decompensated complications, liver failure, and hepatocellular carcinoma—along with poor prognoses, would be inevitable. Therefore, early detection and treatment for PBC are necessary.

## Pathogenesis

Genetic susceptibility and exposomes jointly contribute to immunologic derangement in the onset and progression of PBC [17]. Monozygotic twin pairs exhibit a pairwise concordance of 63% in PBC, surpassing most autoimmune disorders in terms of heritability metrics [18]. Consistently, first-degree relatives demonstrate a high prevalence of 0.72% in PBC [19]. Genome-wide association studies and meta-analysis on PBC have identified a number of genetic susceptibility loci associated with immune dysregulation and inflammation promotion in both Human Leukocyte Antigen Genes (HLA, HLA-II) and non-HLA regions [20–26]. On the other hand, the environmental exposome, such as chemical exposure to xenobiotics [1, 27, 28], and biological exposure, such as *Mycoplasma* and *Escherichia coli* [29–31], could be the culprit in breaking tolerance to the E2 subunit of the mitochondrial Pyruvate Dehydrogenase Complex E2 Component (PDC-E2) via molecular mimicry [28, 32, 33].

The immunopathology of PBC can be generalized as targeted intrahepatic Biliary Epithelial Cell (BEC) injury resulting from immune dysregulation caused by the loss of tolerance to mitochondrial proteins [34, 35]. Circulating Antigen-Presenting Cells (APCs) activated by apoptotic vesicles of BECs facilitate the recruitment of multiple adaptive immune cells into the portal tracts. CD4 + T cells [36], including pro-inflammatory Th1, Th17 cells [37–39], T Follicular Helper Cell (T_FH_) [40, 41], downregulated Regulatory T cells (Treg) and T Follicular Regulatory Cell (T_FR_) cells, as well as CD8 + T cells [42], jointly aggravate small bile duct damages and uncontrolled inflammatory response [43–47]. PDC-E2-specific AMAs are generated and secreted by B cells. Innate immunity, involving NK T cells [48], Mucosal-Associated Invariant T cells (MAIT), Myeloid-Derived Suppressor Cell (MDSC) [49, 50], and pro-inflammatory macrophages [51], also contributes to the liver pathology of PBC. Furthermore, the biliary bicarbonate barrier damage caused by the downregulation of Anion Exchanger 2 (AE2), the primary Cl–/HCO3– transporter on the surface of BECs [52], leads to an increase in extracellular acidity [53, 54], which promotes the production of dissolving toxic hydrophobic acid and increases the sensitivity of apoptosis for BECs [55].

Last but not least, epigenetic changes have been clarified as the intermediary mechanism between environmental and genetic factors [56]. Differentially methylated genes, post-translational modifications of histones, the expression of Non-Coding RNAs (ncRNAs) [57], and telomere dysregulation in BECs [58] have been substantiated in the epigenetic pathogenesis of PBC. Intriguingly, epigenetic changes, such as haploinsufficiency for X chromosomes and skewed expression of X chromosome-linked genes, may also partly account for the female dominance in PBC [59, 60].

## Treatments

The treatment goal is to delay the development of PBC, reflected in improving liver biomarkers, preventing decompensated cirrhosis complications, and relieving symptoms. Here, we discuss the current treatment options:

### First-Line Treatment and Efficacy Assessment

Ursodeoxycholic Acid (UDCA) is the first-line pharmacotherapy approved by the Food and Drug Administration (FDA) since 1994. UDCA, a secondary bile acid, is biosynthesized through gut microbial transformation of chenodeoxycholic acid within the intestinal microenvironment. It is absorbed in the colon and transferred to the circulating bile acid pool. UDCA functions through several mechanisms, such as reducing toxic hydrophobic bile acids, promoting bile secretion and transformation, protecting BECs from apoptosis or endoplasmic reticulum stress-related autophagy, and exerting anti-inflammatory and immunomodulatory effects [61]. Its therapeutic efficacy in ameliorating biomarkers such as Alkaline Phosphatase (ALP), Glutamyl Transpeptidase (GGT), Alanine Aminotransferase (ALT), Aspartate Aminotransferase (AST), Total Bilirubin (TB), Total Cholesterol (TC), Immunoglobulin M (IgM) level [62–64], and delaying histopathological progression to liver cirrhosis [65, 66] has been validated in various clinical studies. UDCA therapy (13 ~ 15 mg·kg^−1^·d^−1^ [67]) exhibited a significant effect on increasing survival time free of liver transplantation [68, 69] and improving long-term prognosis [70, 71]. Although some patients may experience adverse reactions, such as diarrhea, abdominal distension, weight gain, aggravated pruritus, and rarely occurring anaphylaxis [72], UDCA is typically well-tolerated. Unless intolerable, continuous medication is required with the need for dose adjustment and regular follow-up treatment surveillance on body mass, symptom management, liver function tests, and liver fibrosis monitoring. Efficacy evaluation criteria on the biochemical response to UDCA with various timing of assessment after UDCA treatment are exhibited (Table 1). Patients with improved biochemical response exhibit better long-term prognoses [70], underscoring the urgent need for alternative second-line therapies for refractory PBC patients.

**Table 1:** Evaluation criteria on the biochemical response to UDCA

Criteria	Time points of assessment	Standards of response
Binary		
Shanghai [73]	baseline	Mayo score > 6.27, platelet (PLT) count < 132.5 × 10^9^/L
Xi’an [74]	1 month	ALP ≤ 2.5 × ULN, AST ≤ 2 × ULN, and TB ≤ 1 × ULN
Ehime [75]	6 months	≥ 70% decrease from baseline in GGT, or GGT ≤ ULN
Beijing [76]	6 months	ALP ≤ 3 × ULN, TB and/or albumin ≤ ULN
Rochester-I [77]	6 months	ALP < 2 × ULN or updated Mayo score < 4.5
Rochester-II [78]	12 months	ALP ≤ 2 × ULN or TB ≤ 1 mg/dL
Barcelona [79]	12 months	≥ 40% decrease from baseline in ALP, or ALP ≤ ULN
Paris-I [71] (for advanced disease stages: Stage III ~ IV according to Ludwig criteria)	12 months	ALP ≤ 3 × ULN, AST ≤ 2 × ULN, and TB ≤ 1 mg/dL
Paris-II [80] (for early disease stages: Stage I ~ II according to Ludwig or normal TB and albumin levels)	12 months	ALP ≤ 1.5 × ULN, AST ≤ 1.5 × ULN, and TB ≤ 1 mg/dL
Rotterdam [70]	12 months	TB ≤ ULN and/or albumin ≥ LLN
POISE [81]	12 months	ALP < 1.67 × ULN, and ≥ 15% reduction from baseline in ALP, TB ≤ ULN
USA [82]	12 months	TB ≤ 1 mg/dL, ALP < 2 × ULN
Monza [82]	12 months	GGT < 3.2 × ULN or ALP < 2 × ULN
Toronto [83]	24 months	ALP ≤ 1.67 × ULN
Montreal [84]	24 months	AST normalization or improved PHG (porto-hepatic gradient)
Continuous		
UK–PBC score [71]	12 months	Baseline: albumin and PLT count 12 months: TB, ALP, and AST (or ALT) prediction of 5, 10, 15 years survival rates and the response to UDCA
PBC GLOBE score [78]	12 months	Baseline: age; 12 months: TB, ALP, albumin, and PLT count; prediction of 5, 10, and 15 years survival rates and the response to UDCA

*ALP* Alkaline Phosphatase, *ALT* Alanine Aminotransferase, *AST* Aspartate Aminotransferase, *LLN* Lower Limit of Normal, *TB* Total Bilirubin, *UDCA* ursodeoxycholic Ursodeoxycholic Acid

### Second-Line Treatments

Statistically, 40% of the patients respond inadequately to UDCA monotherapy [85], which leads to unrestrained PBC progression and decreased rates of transplant-free survival. Therefore, extra anti-cholestasis therapies, primarily Obeticholic acid (OCA), fibrates (Bezafibrate and Fenofibrate), and Budesonide, are prescribed as second-line treatments in patients with refractory PBC.

#### Obeticholic Acid (OCA)

As a selective agonist, OCA inhibits bile acid synthesis by binding with Farnesoid X Receptor (FXR), whose downstream signals further affect inflammation, metabolism regulation, and the progress of liver fibrosis [86]. It can be used as a bigeminy therapy with UDCA to improve unfavorable biochemical response or as an alternative monotherapy for those who are intolerant to UDCA. It starts at 5 mg/d and increases to 10 mg/d after 6-month treatment based on efficacy and safety response assessment [67], and the main side effect is dose-dependent pruritus with sufficient tolerance [87].

OCA used to be the first conditional-approved second-line therapy for PBC by the FDA and the European Medicines Agency (EMA). In the phase-III POISE clinical trial, compared with the placebo group, the biochemical response rates were 36% (*p* < 0.001, Relative Risk (RR) = 4.85; 95% CI, 2.3–10.23) and 37% (*p* < 0.001, RR = 4.85; 95% CI, 2.3–10.23) higher in the OCA 5–10 mg and 10 mg groups, respectively [81, 88]. Another study demonstrated that the transplant-free survival rate in the OCA group was 7.6% (Hazad Ratio (HR) = 0.29; 95% CI, 0.10–0.83) and 10.8% (HR = 0.30; 95% CI, 0.12–0.75) higher than the external real-world data of GLOBE-PBC cohort and UK-PBC cohort, respectively [89]. Subsequently, the phase-IV COBALT study was designed to assess the effect of OCA on improving clinical outcomes in patients with PBC [90]. Due to lack of efficacy and challenges in patient recruitment, the study was terminated ahead of schedule. Results from the intent-to-treat analysis showed that the incidence of the primary composite endpoint was comparable between the OCA group and the placebo group, at 28.6% and 28.9% (HR = 1.01; 95% CI, 0.68–1.51), respectively. However, when compared with an external control group derived from US medical claims databases, weighted analysis revealed that the incidence of the primary endpoint in the OCA group was 10.4%, lower than the non-OCA group (*p* = 0.001, HR = 0.39; 95% CI, 0.22–0.69). These findings suggest that OCA treatment may reduce the risk of adverse clinical outcomes in PBC patients. In the external real-world HEROES study including 4174 patients with refractory PBC, the OCA group demonstrated a 63% reduction in the risk of adverse outcomes (HR = 0.37, *p* < 0.001) [91]. In other real-world cohort studies from Italy, Canada, and Spain, improvements in biochemical parameters and good tolerability were also observed [92–94]. Due to the clinical benefits of OCA (Ocaliva®) not outweighing its risks, its conditional marketing authorization in the European Union has been formally revoked on November 27, 2024. Although the FDA has not yet revoked the marketing authorization of Obeticholic acid in the USA, OCA was reported to give rise to severe liver decompensation events (hepatic encephalopathy, ascites, esophageal and gastric varices, continuous reduction of PLT [95]). Thus, it is restricted for cirrhotic patients with portal hypertension or manifesting decompensation features [67].

#### Fibrates

Fibrates have been widely used to regulate blood lipids in patients with hyperlipidemia [96]. Recently, off-label utilization of fibrates to enhance biochemical response has been investigated based on its actions on Peroxisome proliferator-Activated Receptor (PPARs).

PPARs are ligand-dependent transcription factors and consist of three subtypes: PPAR-α, PPAR-δ, and PPAR-γ. PPAR-α activation can modulate bile acid metabolism. Fenofibrate functions as a PPAR-α agonist. As a secondary therapeutic option, Fenofibrate has demonstrated consistent efficacy in ameliorating hepatic biomarkers among UDCA-refractory individuals, particularly evidenced by enhanced ALP normalization rates [97]. Further retrospective cohort analyses involving PBC patients with cirrhosis have corroborated its capacity to improve biochemical response and long-term clinical outcomes [98, 99]. Another investigation has further revealed the beneficial effects of Fenofibrate on bile acid homeostasis modulation, specifically through reducing the accumulation of hepatotoxic bile acid species [100]. These studies provide evidence-based support for the potential future use of fenofibrate as an intensive treatment method.

Another widely used fibrate, Bezafibrate, has shown a therapeutic effect by activating all three subtypes of PPARs. Combination treatment of bezafibrate and UDCA remarkably contributes to marked reduction or normalization of ALP in PBC patients who respond insufficiently to UDCA. A meta-analysis incorporating seven randomized controlled trials (*n* = 177 PBC patients) revealed that combination therapy with Bezafibrate and UDCA significantly decreased serum levels of ALP, GGT, IgM, and TC. However, this therapeutic approach demonstrated no statistically significant improvement in pruritus severity [101]. Notably, this finding contrasts with emerging evidence from subsequent clinical investigations. A longitudinal study involving 48 PBC patients with ALP elevation demonstrated both symptomatic relief in pruritus and biochemical improvement (54% ALP normalization rate) in the combination therapy cohort [102]. Furthermore, early therapeutic benefits manifested as decreased ALP levels and attenuated pruritus intensity within 3 months of treatment initiation were reported [103]. Crucially, previous studies consistently identified progressive diminution of therapeutic efficacy correlating with advanced disease staging and cirrhotic progression. Consistent with previous findings, a retrospective study revealed that additional use of bezafibrate significantly reduced UK-PBC and GLOBE risk scores in early-stage PBC cases, prolonging transplant-free survival and improving long-term prognosis [104]. To elucidate prognostic determinants in patients receiving dual therapy, another investigation employing Cox regression analysis of 772 PBC cases identified ALP, TB, albumin, and advanced histological stage as independent predictors of adverse clinical outcomes [105]. In addition, these therapeutic approaches demonstrated diminished therapeutic efficacy in advanced-stage PBC patients, highlighting the critical importance of early diagnosis and timely intervention. The most common adverse reactions of PPAR activators include elevated transaminase levels, gastrointestinal symptoms, and musculoskeletal pain. Additionally, PPAR activators can lead to creatinine elevation, necessitating cautious use in patients with renal impairment [106, 107]. Notably, drug-drug interactions with statins increase the risk of rhabdomyolysis [108]. Real-world study data also demonstrated that 10% of patients develop drug-induced liver injury, warranting close monitoring of biochemical parameters during treatment [109].

### Budesonide

Budesonide has dual agonistic effects on Glucocorticoid and Pregnane X Receptors, which regulate bile acid synthesis, transport, and metabolism. Given its high intrahepatic first-pass effect (90%), it can reduce systemic adverse reactions and related complications of Glucocorticoid. A 24-month prospective cohort study involving 39 patients of mostly early-stage PBC demonstrated that combination therapy with UDCA and Budesonide (3 mg TID) yielded significantly greater improvements in liver enzymes, immunoglobulins, and histopathological evaluation compared to UDCA monotherapy [110]. Another 36-month double-blinded trial also supported the efficacy of Budesonide (9 mg/d) in alleviating liver biomarkers, especially in ALP normalization. However, no particular effects on liver histological improvement and increased risk of portal vein thrombosis related to patients with advanced PBC were observed in the combination group of UDCA and Budesonide [111]. Besides, limited effect on decreasing liver biomarkers after 12-month additional treatment of Budesonide was elucidated, accompanied with aggravating osteoporosis on 22 patients with incomplete response to UDCA [112]. While Budesonide appears clinical potential, its therapeutic efficacy and safety remain controversial, highlighting the need for data from large-scale clinical cohort to establish evidence-based consensus.

### Liver Transplantation

Liver transplantation serves as the sole curative modality in terminal stages of PBC. It is considered when severe, intractable pruritus lasts, or diagnosis of decompensated cirrhosis and the Model for End-Stage Liver Disease (MELD) score > 15 or Mayo risk score > 7.8 are simultaneously satisfied [113, 114]. As an international cohort study on 3902 patients from Europe and North America reported, UDCA has the benefit of prolonging liver transplantation-free survival [68]. Thus, continuous UDCA treatment is needed for post-transplantation patients to prevent and minimize recurrence [115]. A recent retrospective study evaluated the clinical outcomes of 332 international patients with post-transplant recurrent PBC [116] and found that both inadequate response to UDCA and elevated prognostic risk scores demonstrated significant correlations to graft loss and adverse outcomes. Hence, minimizing the recurrence of PBC poses a significant challenge.

### Treatment of Extrahepatic Complications

Patients with PBC commonly experience distressing symptoms such as pruritus and fatigue, significantly impacting their overall well-being. The management of pruritus is part of the essential treatment goal in the risk stratification strategy of PBC patients, which will be elaborated in the subsequent text. Fatigue occurs in about 80% of patients with PBC, significantly impacting their quality of life [117]. Thus far, no pharmaceutical interventions have been permitted for the treatment of fatigue and cognitive symptoms. The current approach primarily involves addressing other potential contributing factors, such as depression, extrahepatic autoimmune diseases, anemia, and sleep disorders, along with mindfulness therapy and ensuring sufficient daily physical activity [118, 119]. The phase-III clinical trial of Seladelpar, a selective PPAR-δ agonist, demonstrated that 55–64% of patients in the Seladelpar group experienced improvements in the PBC-40 fatigue scoring [120]. An open-label prospective study revealed that the combination therapy of UDCA and S-Adenosyl-L- Methionine (SAMe) significantly ameliorated the scores of fatigue, pruritus, and anxiety symptoms in the PBC-40 scale [121]. The novel Gamma-Aminobutyric Acid Type A (GABA-A) receptor-modulating steroid antagonist, Golexanolone, has demonstrated preliminary efficacy and safety in a bile duct-ligated rat model and a phase-Ib/II pilot trial of cirrhosis patients, respectively, which exhibits significant therapeutic potential for managing fatigue, motor, and cognitive impairments in PBC patients [122, 123]. Artificial tears and cyclosporin eye drops benefit ocular dryness [124]. Maintaining good oral hygiene and utilizing saliva substitutes are helpful to maintain oral moisture. Choline receptor agonists like Pilocarpine or Cevimeline are also deemed efficacious for oral dryness [125]. PBC-associated metabolic bone disease is characterized by bone demineralization and the development of osteoporosis [126], where Bisphosphonates, vitamin A, vitamin D, and Calcium supplementation are helpful [127]. For PBC patients with hypercholesterolemia, lipid-lowering medications are only considered for those with metabolic syndrome and cardiovascular risk factors [128].

### Novel Therapeutic Options

A number of pharmacological agents targeting cholestasis pathways are now in advanced developmental stages. These novel therapies, complemented by breakthroughs in immunomodulatory interventions and stem cell-based therapy (Table 2), are projected to substantially broaden the therapeutic armamentarium for PBC, thereby enabling personalized treatment paradigms in clinical hepatology.

**Table 2:** Clinical and preclinical trials of drugs for the treatment of PBC

Drug	Mechanism of action	Publication	Type of trials	Findings
Fenofibrate	PPAR-α agonist	Am J Gastroenterol. 2023 [129]	12-month randomized-parallel, open-label trial (NCT02823353)	Met Barcelona (UDCA-Fenofibrate combination group: 81.4% vs. UDCA monotherapy: 64.3%)
Bezafibrate	Triple PPAR-α/δ/γ agonist	Gastroenterology. 2021 [130]	Double-blind, randomized, placebo-controlled trial (**FITCH**, NCT02701166)	≥ 50% reduction of moderate-to-severe pruritus (Bezafibrate 400 mg OD: 55% vs. 11% in the placebo group)
Elafibranor (Iqirvo®)	Dual PPAR-α/δ agonist	N Engl J Med. 2024 [131]	Phase-III, 52-week, double-blind, placebo-controlled trial (**ELATIVE**, NCT04526665)	Met POISE (Elafibranor 80 mg OD: 51% vs. 4% in the placebo group) ALP renormalization rate (15% vs. 0)
		J Hepatol. 2021 [132]	Phase-II, 12-week, double-blind trial (NCT03124108)	Met POISE (elafibranor 80 mg: 67%, 120 mg: 79%); decrease in ALP, GGT
Seladelpar (Livdelzi®)	PPAR-δ agonist	N Engl J Med. 2024 [120]	Phase-III, 12-month, double-blind, placebo-controlled trial (**RESPONSE**, NCT04620733)	Met POISE (seladelpar 10 mg: 61.7% vs. 20.0% in the placebo group) ALP renormalization (25.0% vs. 0%) reduction in the score on the pruritus NRS
		Hepatology. 2024 [133]	Phase-II, open-label study (NCT02955602)	Decreases in serum IL-31, bile acids, and pruritus NRS
		Hepatology. 2023 [134]	Phase-III, 3-month, double-blind, randomized-controlled trial (early-terminated **ENHANCE**, NCT03602560)	Met POISE (Seladelpar"5aliensec2 Treatment of Extrahepatic Complications"mg: 57.1%, 10 mg: 78.2%) ALP renormalization rate (5.4% and 27.3%) Reduction in NRS
		J Hepatol. 2022 [135]	Phase-II, 52-week, dose-ranging, open-label study (NCT02955602)	Met POISE (Seladelpar"2aliensec1 Second-Line Treatments"mg, 5 mg, 10 mg: 64%, 53%, 67%) ALP renormalization rate (9%, 13%, and 33% in the 2 mg, 5 mg, and 10 mg groups)
		Lancet Gastroenterol Hepatol. 2017 [136]	Phase-II, 12-week, double-blind, placebo-controlled trial (NCT02609048)	Decreases from baselines in ALP (2%, 53%, 63% in placebo, seladelpar 50 mg, 200 mg groups); associated with increases in ALT
Saroglitazar	Dual PPAR-α/γ agonist	J Hepatol. 2022 [137]	Phase-II, 16-week, double-blind, proof-of-concept trial (NCT03112681)	Reduction in ALP (saroglitazar 2 mg: 49%, 4 mg: 51%, and 3% in the placebo group)
Setanaxib	NOX1/4 inhibitor	Hepatol Commun. 2023 [138]	Phase-II, 24-week, double-blind, randomized-controlled trial (NCT03226067)	Reductions in fatigue and other PBC-40 domains except itch
		Liver Int. 2023 [139]		Decreases in ALP and GGT and potential anti-fibrotic effect
Tropifexor	FXR agonist	JHEP Rep. 2022 [140]	Phase-II, double-blind, placebo-controlled trial (NCT02516605)	Decrease in GGT
Rituximab	Anti-CD20 monoclonal antibody	Hepatology. 2012 [141]	Open-label study (NCT00364819)	Decreases in IgM and ALP; decreases in memory B-cell and T-cell frequencies
	Anti-CD20 monoclonal antibody	J Gastroenterol. 2013 [142]	Open-label pilot study	B cell depletion; decreases in ALP, IgM, and AMAs
Mesenchymal stem cell transplantation	Increasing Gal-9 expression	J Gastroenterol Hepatol. 2013 [143]	Single-arm trial (NCT01662973)	Improvement in fatigue and pruritus; decreases in ALP and GGT

*ALP* Alkaline Phosphatase, *ALT* Alanine Aminotransferase, *GGT* Glutamyl transpeptidase, *NRS* the pruritus numerical rating scale, *MWDI *Mean Worst Daily Itch, *UDCA* Ursodeoxycholic Acid, *FXR* Farnesoid X Receptor, *PPAR* Peroxisome proliferator-Activated Receptor, *NOX*  Nicotinamide Adenine Dinucletide Phosphate Oxidase, *JAK* Janus  Kinase

#### Elafibranor

Elafibranor (Iqirvo®) is a dual PPAR-α/δ agonist that has received accelerated approval by the FDA and EMA as the first-in-class PPAR-targeted therapy for PBC in 2024 [144, 145]. It can be applied as a monotherapy or with UDCA, determined by response or tolerance to UDCA. In the 52-week phase-III ELATIVE trial, the proportion of patients in the Elafibranor 80 mg qd group achieving the POISE biochemical response criteria was 47% (*p* < 0.001; 95% CI, 32 to 57) higher than that in the placebo group, and 15% (*p* = 0.002; 95% CI, 6 to 23) of patients in the Elafibranor group achieved ALP normalization [131]. The predominant adverse reactions observed were gastrointestinal symptoms, including nausea, emesis, stomachache, and diarrhea.

#### Seladelpar

Seladelpar (Livdelzi®) functions as a selective agonist for PPAR-δ that has received accelerated approval by the FDA as an effective second-line treatment or alternative monotherapy for UDCA intolerant patients [146]. A phase-II clinical trial has substantiated the enduring dose-dependent efficacy of Seladelpar at low doses in diminishing biomarkers of cholestasis, mainly ALP, in UDCA-intolerant or incomplete responders [135]. In the phase-III RESPONSE trial, the safety and efficacy in the Seladelpar group have been verified, which demonstrated a higher biochemical response rate, ALP renormalizing rate, and a significant improvement in baseline moderate-to-severe pruritus symptoms along with generally mild-to-moderate adverse events (including headache, nausea, abdominal pain) [120]. In the phase-III, randomized placebo-controlled ENHANCE trial, a notable reduction of transaminase was also observed in the Seladelpar group, and pruritus improvement was consistent with the previous findings [134]. The mechanism by which Seladelpar alleviates pruritus is associated with reduced serum IL-31 levels and bile acid concentrations [133]. Seladelpar has also been proven to improve Metabolic-associated Steatohepatitis (MASH) and protect from liver fibrosis, which may benefit obese PBC patients [147, 148]. Regulatory assessment for the use of Seladelpar in treating PBC is underway in the EU and the UK.

#### Others

A number of new pharmaceuticals targeting various mechanisms are currently being evaluated in clinical trials. Saroglitazar, a dual agonist for PPAR-α/γ, could significantly decrease ALP [137]. However, further research is required to determine the safe dosage for preventing increased liver transaminases. Tropifexor is a novel FXR agonist that has demonstrated beneficial effects on reducing hepatic cholestasis markers, inhibiting inflammatory cell infiltration, and liver fibrosis, while it may induce pruritus [140, 149]. Moreover, setanaxib is a selective NADPH Oxidase enzyme subtypes 1 and 4 (NOX1/4) inhibitor with anti-cholestasis and anti-fibrotic effects, as reflected in ALP, liver stiffness, fatigue, and cholestasis improvement in a phase-II trial [139]. Researchers are conscientiously looking for alternative options to treat patients with refractory PBC.

#### Immunotherapies

The distinct liver-specific autoimmune features of PBC are characterized by immune-mediated bile duct injury and chronic cholestasis, manifested by AMAs (90% of patients) and specific antinuclear antibodies (anti-sp100, anti-gp210 in 10 ~ 20% of patients), elevated proinflammatory cytokines, and subsequent cellular and humoral immune responses [150–152]. The successful preclinical investigations in various animal models of PBC (Table 3) [153] and clinical trial data have elucidated immune-targeted modalities addressing pathogenic mechanisms, and anti-inflammatory interventions have demonstrated optimal clinical utility during initial disease phases [151].

**Table 3:** Preclinical studies on immunotherapies for PBC

Drug	Mechanism of action	Publication	Type of animal experiment	Findings
Ruxolitinib	JAK inhibitor	Cell Mol Immunol. 2022 [154]	ARE Del ± mice	Reduced portal inflammation and bile duct damage, lower AMA levels, reduced liver and peritoneal macrophages and polarized from M1 to M2
Baricitinib	JAK inhibitor	Nat Commun.2024 [155]	Experiment on 2OA-BSA mice	Histological improvements; reduced inflammatory cytokines; decreased Th1-like cell infiltration
PD-1 targeting CAR-T cells	Selective CD8 + T cell eliminator	Nat Commun. 2024 [156]	Experiment on Il12b^−/−^Il2ra^−/−^ mice (DKO mice)	Effective alleviation of PBC
PD-1 targeting CAR-T cells	Selective CD8 + T cell eliminator	Adv Sci (Weinh). 2024 [157]	Experiment on dnTGFβRII Aire mice	Effective alleviation of PBC
TKM-011	Anti-CD20 monoclonal antibody	Front Immunol. 2018 [158]	Experiment on dnTGFβRII mice expressing hCD20 and human Fcγ receptors (hFcγRs)	Reduction in portal inflammation and CD8 + T cells; decreases in AMAs

*JAK* Janus Kinase, *AMA* Anti-Mitochondrial Antibodies, *Th1* Helper T cell 1, *CAR-T* Chimeric Antigen Receptor T cell, *TGF-βR* Transforming Growth factor - β Receptor, *PD-1* Programmed Cell Death Protein 1

##### Targeting T cells

Pro-inflammatory Th1 cell suppression has demonstrated great preliminary potential in alleviating cholestasis in mouse models of PBC. The Janus Kinase/Signal Transducer and Activator of Transcription (JAK/STAT) signaling pathway in liver resident Th1-like cells was significantly activated in patients with PBC [155] and the 2OA-BSA mouse model. The JAK inhibitor, Baricitinib, has demonstrated marked histological improvements, reduced levels of inflammatory cytokines, and decreased Th1-like cell infiltration in the 2OA-BSA mouse model [155]. In the ARE-induced PBC murine model, therapeutic intervention with the JAK inhibitor Ruxolitinib demonstrated multifaceted immunomodulatory effects. Compared with vehicle-treated controls, the treatment group exhibited marked reductions in hepatic CD4 + and CD8 + T lymphocyte populations. Concurrently, histopathological evaluation revealed attenuated portal inflammation and improved bile duct integrity, accompanied by a significant decrease in serum AMA titers. Notably, macrophage redistribution was observed with concomitant M2 polarization predominance over pro-inflammatory M1 phenotypes [154]. Further validation of the safety and efficacy of JAK inhibitors is required in larger clinical cohorts in patients with incomplete response to UDCA.

Alleviating autoimmune response by supplementing antigen-specific Treg cells or enhancing their functions in treating PBC was also explored. Recently, a research team has successfully developed an Engineered Treg Cell (EngTreg) expressing T cell Receptors (TCRs) for a novel PDC-E2 epitope that specifically inhibits PDC-E2-specific polyclonal CD4 + conventional T cells in patients with PBC. This PDC-E2 epitope is associated with the HLA-DRB4*01:01 allele and is present in over 60% of PBC patients, suggesting its potential as a therapeutic target for PBC. Strikingly, PDC-E2-specific EngTregs effectively suppressed polyclonal PDC-E2-specific Teff derived from patients with PBC[159]. Furthermore, using a biodegradable nanoparticle, ImmTOR, encapsulated with Rapamycin in combination with the engineered IL-2 mutein, which can significantly increase the number and durability of Tregs in the NOD.c3c4 mouse model of PBC, markedly reduced the expansion of effector immune cells, suppressed immune response, and demonstrated good safety and efficacy[160].

CD103 + Tissue Resident Memory T(TRM) cells are the dominant CD8 + T cell population and are localized near the biliary ducts in patients with PBC. T cell-targeted therapies were investigated in various animal models of PBC. In the 2OA-BSA mouse model, inhibition of the Nucleoside Diphosphate X Hydrolase 1 (NUDT1) eliminated CD103 + TRM cells and attenuated cholangitis [161]. In the Il12b^−/−^Il2ra^−/−^ mouse model, treatment with a specific Programmed Cell Death Protein 1 (PD-1) -targeting Chimeric antigen Receptor T (CAR-T) cell selectively depleted liver CD8 + TRM cells and alleviated autoimmune cholangitis [156]. Clonal expansion of functionally activated PD‐1 + CD8 + T cells in the livers of dnTGFβRII Aire^−/−^ mice, along with increased levels of granzyme B and perforin -1, partly accounted for the CD8 + T - induced pyro-death of hepatocytes. Administration of PD-1 CAR-T cells was able to clear CD8 + T cells in these studies [157]. These findings warrant further development of T cell-targeted therapy in PBC and expand the target of CAR-T cells to treat PBC [162].

##### Targeting B cells

Anti-CD20 antibodies can selectively knock out the B lymphocyte subtype, alleviating lymphoma and autoimmune diseases. Anti-CD20 monoclonal antibody, Rituximab, has demonstrated preliminary clinical effects in reducing the numbers of memory B and T cells accompanied by limited improvement in liver biomarkers such as ALP and AMAs in patients with persistent biochemical abnormalities to UDCA [141, 142]. dnTGF-βRII mice treated with humanized anti-CD20 antibody TKM-011 showed reduced bile duct inflammation, CD8 + T cell infiltrations in the liver, and decreased AMAs [158]. The B-Cell Activating Factor of the TNF family (BAFF) functions through the BAFF Receptor (BAFFR) to enhance B cell survival, thereby facilitating immunoglobulin class switching and recombination. Recent studies have indicated a positive correlation between serum BAFF levels, AMA titers, and the frequency of circulating plasma blasts, indicating that BAFF may be involved in the initiation and advancement of PBC [163]. Intriguingly, the baseline serum levels of BAFF are inversely correlated with the duration of B cell depletion. A close correlation between decreased BAFF and symptom improvement has been observed in PBC patients [164]. Using a dual-pronged approach, Zhang et al. demonstrated that the combination of anti-BAFF and anti-CD20 significantly reduced serum AMA levels as well as B cell populations in peripheral blood and tissues. This treatment also markedly ameliorated autoimmune cholangitis in the female ARE-Del mouse model of PBC [165].

#### Mesenchymal Stem Cell Transfusion

A single-arm clinical trial on seven PBC patients, which failed to achieve complete biochemical responses, reported that Umbilical Cord-Derived Mesenchymal Stem Cell (UC-MSC) transfusion significantly improved symptoms like fatigue and pruritus and reduced cholestasis biomarkers like ALP and GGT [143]. Multi-center randomized-controlled clinical trials with larger cohorts are needed to validate this approach.

## When and How to Add Intensive Therapies Beyond First-Line Treatment?

To further improve the prognosis of PBC patients, an intensive risk stratification strategy aiming at more stringent treatment goals and earlier therapeutic response evaluation in clinical practice is imperative. Tailored treatments should be methodologically designed for high-risk patients.

### Intensive Therapies Based on more Stringent Goals

#### More Rigorous Histological Monitoring: PBC-AIH Overlaps Identification

Although liver biopsy is routine for pretreatment risk stratification [17], it is not a necessity for the diagnosis of autoantibody-positive PBC, except for two specific situations. Firstly, for cholestasis patients with excluded obstruction of large bile ducts by imaging examination and negative PBC-specific autoantibodies, liver biopsy could aid in the differential diagnosis of cholestatic diseases stemming from alternative etiologies or those characterized by bile duct injury, lymphocyte aggregation, and granuloma as pathological manifestations, for instance, Metabolic Associated Fatty Liver Diseases (MAFLD), Drug-Induced Liver Damage (DILI), and PBC-AIH overlap syndrome [166]. Secondly, for individuals with suboptimal biochemical response, particularly those exhibiting significant elevations in ALT and/or IgG levels which are irregular in PBC (especially those with ALT > 5 × ULN or IgG ≥ 1.3 × ULN), liver biopsy should be considered an initial step to assess the presence of moderate-to-severe interface hepatitis to exclude PBC-AIH overlap syndrome [167]. Although PBC-AIH overlap syndrome is found in only 1 ~ 3% of PBC patients [168], up to 25% exhibit features of interface hepatitis with diverse severities in patients with PBC who undergo liver biopsy [169]. Despite the pathological feature of PBC being nonsuppurative granulomatous inflammation of the intrahepatic interlobular bile ducts, about 25% of PBC patients are accompanied by various levels of interface hepatitis pathologically [170].

For PBC patients with moderate-to-severe interface hepatitis, combined medication of UDCA and immunosuppressants may have therapeutic benefits [171, 172]. However, the long-term use of immunosuppressants may lead to adverse side effects [173]. Moreover, not every single patient can benefit from steroid treatment. For young patients with elevated ALT/AST and interface hepatitis, which often represents a more aggressive phenotype of PBC, second-line therapies should be included before immunosuppressants are considered [166].

Precise identification of patients most likely to have fibrosis reversal through combined treatment of UDCA and immunosuppressants is of great clinical importance. Previous studies have found that the Paris criteria might lack sensitivity for steroid responsiveness. A new threshold of IgG ≥ 1.3 × ULN, which has increased the sensitivity for steroid responsiveness by 50%, was proposed [174]. Another multicenter retrospective study has developed an efficacy-predictive score of combined therapy of UDCA and immunosuppressants, the Fibrosis Regression Score (FRS), based on the thresholds of baseline IgG > 1.3 × ULN and ALP > 2.4 × ULN. It can effectively identify specific PBC patients with moderate-to-severe interface hepatitis who can benefit from combined immunosuppressants (Prednisone, Azathioprine (AZA), Mycophenolate Mofetil (MMF)), as evidenced by significant improvement in histological fibrosis regression (rate increased from 6.3% to 52.4%), biomarkers, survival rates, and long-term prognoses [175]. This scoring system provides clinicians with a powerful tool to assess treatment response more precisely case by case, leading to the optimization of personalized medicine.

Severe interface hepatitis serves as an independent risk factor for UDCA nonresponse and contributes to a correspondingly increased rate of liver transplantation [71]. Hence, a clear diagnosis is needed first and foremost. The endorsed Paris criteria [167]  suggest that the PBC-AIH overlap syndrome can be diagnosed when liver histology shows moderate-to-severe interface hepatitis with at least one of the two diagnostic standards of AIH being satisfied simultaneously for PBC patients: (1) serum ALT ≥ 5 × ULN and (2) serum IgG ≥ 2 × ULN or serum Anti-Smooth Muscle Antibodies (ASMAs) react positively.

The treatment strategy of PBC-AIH overlap syndrome and the factors related to treatment response have yet to be unified, and the empirical treatment is the primary treatment [7]. Some studies have shown that UDCA monotherapy itself may improve efficacy and safety in treating PBC-AIH overlapping syndrome [176, 177]. However, other studies have suggested that combined medication of UDCA and immunosuppressants improved histology and liver biomarkers more than UDCA monotherapy [173, 178]. It was noted in the European Association for the Study of the Liver (EASL) guidelines that patients exhibiting typical features of PBC and AIH may also benefit from immunosuppressive therapy in conjunction with UDCA [113]. The administration of immunosuppressants for PBC-AIH overlap syndrome is mainly based on the management of Autoimmune Hepatitis (AIH): Predisone, in conjunction with AZA: Predisone is utilized to induce remission, providing rapid symptom relief and achieving a biochemical response; AZA serves to maintain remission by reducing the dosage of Prednisone and minimizing the risk of corticosteroid-related adverse effects. Patients exhibiting inadequate response or contraindications to steroid hormones and AZA may contemplate transitioning to second-line immunosuppressants, such as MMF, Cyclosporine A, or tacrolimus [179–181].

#### Stricter Goal of Biochemical Response: ALP Normalization

ALP is an independent predictor of PBC prognosis. Although current evaluation criteria have not required ALP renormalization, several studies have found that ALP renormalization should be a goal in the optimal management of high-risk individuals with PBC. In patients with qualified biochemical response under the current criteria, there still exists a high incidence (17.0 per 1000) of poor outcome if the ALP level is abnormal, especially in younger patients with advanced fibrosis [182]. Intensive treatment must be applied to these patients to improve complication-free survival.

A cohort study illustrated that the 10-year survival rate of patients with ALP ≤ 1 × ULN was 93.2% compared to 86.1% in those with ALP 1 ~ 1.67 × ULN, along with an increase of 11% when the TB standard was raised from ≤ ULN to ≤ 0.6 × ULN as well [183]. In another retrospective study, patients with continuous increases in ALP from 1.1 to 1.5 × ULN have poorer outcomes than those with normal ALP [182]. In a 10-year follow-up study of patients with only positive AMAs and normal liver biomarkers, a median period of 17.8 years free of liver fibrosis was observed [184], confirming the clinical benefit of ALP normalization. Consequently, ALP normalization and more stringent TB can be regarded as improved goals for early clinical risk stratification.

ALP normalization as a therapeutic target has been verified as a realizable goal in multiple clinical trials. In the phase-III ELATIVE clinical trial of Elafibranor, 51% of the experimental group met the primary endpoint of an adequate response,  and only 15% of the patients reached ALP normalization after 52-week treatment with Elafibranor[131]. In a phase-III clinical trial of Seladelpar at 10 mg, 61.7% of the Seladelpar group satisfied the POISE standard in 12 months, and 25% of them achieved ALP normalization [120]. In another 3-month phase-III ENHANCE trial, the complete response rate and ALP normalization rate of PBC patients treated with Seladelpar were 78.2% and 27.3%, respectively [134].

A few combination regimens have shown great efficacy in ALP normalization. A study on the initial combination of UDCA and fenofibrate has also exhibited significant improvement in the biochemical response rate of 17.1% after 1 month of treatment. In comparison, the ALP recovery rate rose from 40 to 62%, with no increase in the rate of adverse events [129]. This study on the combination therapy of UDCA and fenofibrate indicated it is safe and effective for patients with refractory PBC and cirrhotic patients with incomplete response, which provided timely initiation of second-line treatment for mitigating disease progression. In a multicenter, retrospective cohort study aimed at determining the beneficial effects of triple therapy of UDCA, OCA, and fibrates in patients with hard-to-treat PBC, an Odds Ratio (OR) of ALP normalization of 3.4 (95% CI 1.4–8.2) has been demonstrated, along with biochemical relief and pruritus improvement [185]. Whether pursuing the therapeutic goal of biochemical normalization is cost-effective still needs to be followed up and validated with further clinical trials to determine the ratio and prognoses of patients who meet this goal [186].

#### Stricter Symptom Management: Pruritus Control

Pruritus manifested in as many as 70% of patients with PBC, which significantly impairs the ability to focus on daily tasks and may even result in uncontrollable scratching and bleeding, fatigue, insomnia [187], and depression [188]. Enhancing life quality through targeted management strategies has been underscored as an effective way to alleviate symptoms like pruritus [189, 190]. Fundamental skincare is suitable for all patients. The initial pharmacotherapy for mild pruritus is Cholestyramine (4 ~ 16 g/day) [191], whereas its tolerability is limited for causing adverse reactions such as nausea, abdominal distension, and constipation. Rifampicin (150 ~ 300 mg BID), as an alternative second-line medication [192], has the potential to induce hepatic injury, hemolytic anemia, and renal impairment, as well as interactions with other pharmaceuticals [193], requiring close surveillance of liver tests.

Apart from conventional therapeutic options outlined in current guidelines, Bezafibrate has demonstrated efficacy in ameliorating itchiness among individuals with PBC across multiple studies [102, 130, 194]. Notably, a double-blinded randomized-controlled trial has revealed substantial relief from cholestatic-related itching, including that associated with PBC, providing compelling clinical evidence supporting the use of Bezafibrate for managing pruritic manifestations in PBC [195].

In addition, several prior clinical studies have shown the dose-dependent effectiveness of the PPAR-δ agonist seladelpar in alleviating pruritus symptoms among patients with PBC [120, 133, 134]. Furthermore, another class of promising drugs, inhibitors of Ideal Bile Acid Transporters (IBATs), operates by decreasing the reabsorption of bile acids, reducing enterohepatic circulation, and consequently alleviating the detrimental impacts of bile acids on hepatic cells [196, 197]. The efficacy of IBATs inhibitors in the treatment of cholestatic pruritus has been preliminarily demonstrated (Table 4), even though diarrhea is ineluctable. In the phase-IIb GLIMMER trial of Linerixibat, a specific IBAT inhibitor, a dosage of 40 mg has shown better relief in pruritus [198]. The other two members in the IBATs inhibitor family, Maralixibat and Odevixibat, have previously been observed to reduce pruritus and bile acid levels in patients with Progressive Familial Introhepatic Cholestasis (PFIC) and chronic cholestasis caused by Alagille syndrome [148–151]. However, no significant difference was found in pruritus relief between the Maralixibat group and the placebo group in a phase-II clinical trial of Maralixibat for PBC [152]. Although the efficacy of Odevixibat in PBC remains to be investigated through clinical trials, its potential therapeutic effect should not be underestimated. This phase-III GLISTEN trial of Linerixibat (NCT04950127) and clinical trials on other potential IBAT inhibitors, such as Volixibat (NCT05050136), are currently in progress to identify potential targeted therapies to alleviate pruritus in patients with PBC.

**Table 4:** IBATs inhibitors for the treatment of cholestatic pruritus

Mechanism of action	Drug	Disease	Publication	Type of trial	Findings
IBAT inhibitor	Linerixibat	PBC	Clin Gastroenterol Hepatol. 2023 [198]	Phase-IIb, dose-ranging, randomized-controlled trial (**GLIMMER**, NCT02966834)	Dose-dependent reduction in MWDI score
	Maralixibat	PFIC	Lancet Gastroenterol Hepatol. 2024 [199]	Phase-III, randomized, double-blind, placebo-controlled trial (**MARCH-PFIC**, NCT03905330)	Reductions in pruritus and serum bile salts
		Alagille syndrome	Lancet. 2021 [200]	Phase-IIb, placebo-controlled, randomized study (**ICONIC**, NCT02160782)	Improvements in serum bile salts and pruritus scores
		PBC	Hepatol Commun. 2019 [201]	Phase-II, 13‐week, randomized, double‐blind, placebo‐controlled trial (NCT01904058)	No significant difference in pruritus relief
	Odevixibat	PFIC	Lancet Gastroenterol Hepatol. 2022 [202]	Phase-III, randomized, placebo-controlled trial (NCT03566238)	Reductions in pruritus and serum bile salts; with good tolerability
		Alagille syndrome	Lancet Gastroenterol Hepatol. 2024 [203]	Phase-III, double-blind, randomized, placebo-controlled trial (**ASSERT,** NCT04674761)	Improvements in pruritus and serum bile acid levels
	Volixibat	Non-alcoholic steatohepatitis	J Hepatol. 2020 [204]	Phase-II, 24-week terminated, double-blind, randomized trial (NCT02787304)	No therapeutic effect

*IBAT*  Ideal Bile Acid Transporters, *PFIC*  Progressive Familial Introhepatic Cholestasis

#### Stricter Liver Fibrosis Management: Liver Stiffness Stratification

Hepatic fibrosis and cirrhosis contribute significantly to the progression of PBC and adverse events. Risk assessment and stratification based on liver stiffness are important for PBC patients in monitoring the progression of fibrosis and decompensated events [205]. Changes in the degree of cirrhosis also contribute to reflecting the dynamic efficacy of the treatment.

Although liver biopsy is the “golden standard” for the diagnosis of liver fibrosis [206], it is limited in clinical application because it is an invasive procedure with poor patient acceptance [207]. Hence, alternative noninvasive diagnostic methods are required to monitor the progression of liver fibrosis. Several noninvasive diagnostic models have been recognized as practical tools in diagnosing liver fibrosis, such as APRI [208], the fibrosis index based on the four factors (FIB-4), and AST/ALT ratio (AAR) [209]. Moreover, studies have shown that Vibration-Controlled Transient Elastography (VCTE) has better diagnostic accuracy for PBC patients than previous noninvasive diagnostic models [210, 211].

 Liver Stiffness Measurement (LSM) based on ultrasound and VCTE have efficiently predicted clinical outcomes in PBC patients, especially those with advanced PBC [212]. For instance, a study has identified that LSM > 12.1 kPa can serve as a threshold for predicting the risk of esophageal and gastric variceal bleeding in patients with PBC [213]. Subsequently, LSM is advantageous for further stratified management of fibrotic patients. A multicenter study included 167 patients who received initial treatment for PBC and utilized LSM ≤ 6.5 kPa and > 11.0 kPa as the diagnostic criteria for early-stage and advanced-stage fibrosis, with positive and negative predictive values at 0.89 and 0.94, respectively [214]. Another international multicenter, large-scale retrospective cohort study including 3985 individuals indicated that patients could be stratified into low, moderate, and high-risk groups according to liver stiffness thresholds of 8 kPa and 15 kPa, where 40% of the patients are of medium-to-high risks [212]. Another international retrospective cohort research focused on 3078 PBC patients with LSM has been carried out. It was determined that the relative fluctuations in LSM were notably correlated with the risk of malignant clinical events [215]. These studies have found that LSM serves as a powerful predictor of clinical outcomes and holds significance as an alternative endpoint for assessing clinical benefit in PBC. A recent study also revealed that the 5 kPa grading method (LSM, 10–15–20–25 kPa) proposed in the Baveno VII consensus equally applies to PBC patients. Baseline LSM thresholds of 10 kPa and 15 kPa could stratify patients with PBC into low, moderate, and high-risk categories to diagnose compensated Advanced Chronic Liver Diseases (cACLD). Furthermore, an increase in clinical liver stiffness is positively correlated with the risk of PBC progression, serving as a more precise indication of disease advancement and long-term prognosis [216].

Timely anti-fibrotic therapy has a vital impact on delaying the fibrotic process and improving the prognosis of patients. OCA (5 ~ 10 mg/day) can be considered on a case-by-case basis for patients with conpensated cirrhosis who have partial response to UDCA when it necessitates close monitoring of disease progression. However, in those with decompensated cirrhosis, as well as in those with coagulation abnormalities and persistent thrombocytopenia, OCA is contraindicated [7]. An animal study demonstrated that OCA, along with apoptosis inhibitor treatment, alleviated liver fibrosis in CCL4 treated mice and thereby underscore the anti-fibrotic effect of OCA [217]. Another study on the efficacy and safety of Fenofibrate in cirrhotic patients inadequately responding to UDCA reported that Fenofibrate effectively improved ALP and serum lipid profiles, reduced UK-PBC scores, and maintained stable ALT, AST, TB, and renal function indexes during treatment [99], which shows the great potential of Fenofibrate in the treatment of liver cirrhosis. In a 24-week phase-II clinical trial of Setanaxib, biochemical remission was observed in the experimental group, although the anti-fibrotic potential still needs to be verified in more extensive trials [139]. Additionally, there was also a tendency to relieve fatigue [138].

### Intensive Therapies Based on Earlier Efficacy Assessments

The current clinical management of PBC could be summarized as the “wait‑to‑fail” strategy, which represents patients showing incomplete reactivity to UDCA by assessment requiring extra second-line medication [218]. Various classifiers (Table 1) are used to determine biochemical response to treatment with UDCA, among which the conventional timepoint is 1 year. Patients with complete biochemical response have excellent prognoses, while loss of biochemical response at any time point is correlated to an increased risk of liver transplantation or liver-related death in PBC patients [219]. A research group analyzing a PBC cohort revealed that 41% of endpoint events (death, liver transplantation, and cirrhosis decompensation) occurred within 2 years after diagnosis [74], highlighting the necessity of early individualized intensive therapy for PBC patients.

Long-term biochemical reactivity to UDCA could be precisely anticipated through pretreatment assessment grounded in baseline clinical data to subsequently maximize the benefit from additional second-line therapy as soon as possible. The predictive model for pretreatment assessment developed by Carbone et al. [220] identified that elevated levels of ALP and TB reduced transaminase activity, younger age at onset, prolonged treatment delay, and more rapid deterioration of ALP at baseline were correlated with diminished responsiveness to UDCA therapy. It was confirmed in an external cohort with a good subject Area Under the operating Curve (AUROC) value (0.83). While further validation in clinical practice is necessary, this pretreatment prediction model establishes a basis for early risk stratification in PBC. Another machine learning algorithm has been developed and validated to predict treatment response using pretreatment data, including three important variables: the levels of TB, TP, and ALT, which has an AUC of 0.856 in the test set [221]. It has demonstrated the efficacy of identifying patients requiring additional treatment to improve prognosis.

Early identification and more effective methods for recognizing high-risk patients represent a crucial advancement in optimizing response assessment criteria. In a retrospective study, a total of 569 patients diagnosed with PBC were monitored for years, and a new early risk-stratification standard (ALP ≤ 2.5 × ULN, AST ≤ 2 × ULN, TB ≤ 1 × ULN) after 1-month UDCA treatment was established, which was referred to as the “Xi’an Standard” [74]. The 5-year survival rate without adverse events was 97% in the responding group, significantly higher than 64% in the non-responding patients. Besides, the Xi’an criterion demonstrates efficacy equivalent to existing international standards in assessing low-risk patients while offering a distinct advantage in evaluating medium-to-high-risk patients with rapidly progressing PBC (training cohort, 82%; validation cohort, 91%). It is the first rapid and dynamic evaluation system for UDCA treatment of PBC patients that provides an attempt to enable timely intervention for non-responsive patients and has significant meaning for enhancing long-term prognosis.

However, the ideal time point for the early assessment of patient response is neither fixed nor uniformly applicable across all individuals. Instead, it depends on a multitude of personalized factors, including histological presentations, coexisting complications, and the success of early diagnosis. LSM has been validated as a reliable method for predicting the risks of portal hypertension, decompensated cirrhosis, and liver-related mortality in diverse liver diseases. Consequently, during the evaluation of treatment response, conducting risk stratification grounded in noninvasive examinations (LSM particularly) and assessing the management of complications are indispensable steps. These measures are essential for tailoring the most optimal treatment for each patient, ensuring individualized and effective care.

## Summary

**Fig. 1:**
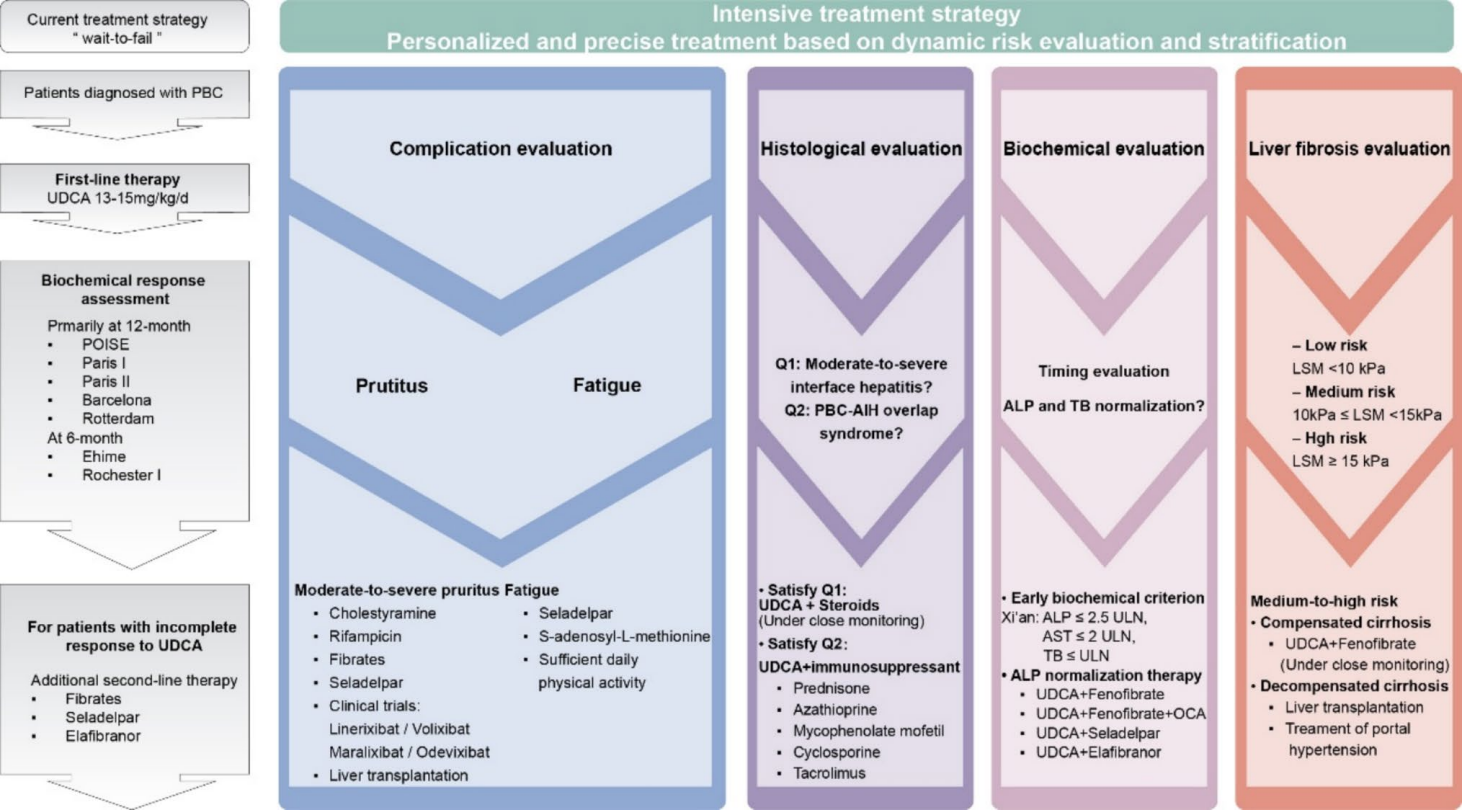
An intensive therapeutic strategy in PBC

The current conventional treatment approach for PBC follows a “wait-to-fail” strategy, primarily assessed when a standardized one-year UDCA treatment is accomplished. Approximately 40% of the patients exhibit inadequate response to UDCA monotherapy; incomplete response to UDCA, as well as cirrhosis, is the main risk factor for PBC complications. Delayed evaluation could lead to missed opportunities for timely intervention, ultimately contributing to disease progression. Hence, two issues must be addressed for this segment of the patients responding inadequately to UDCA. On one hand, the availability of effective additional treatment options must be enriched. The future therapeutic direction for PBC is likely to involve combination therapy with drugs targeting multiple pathways. Clinical research on new medications targeting diverse pathways has yielded preliminary safety and efficacy. On the other hand, the standard of intensive therapies for PBC needs to be harmonized to improve the prognosis of PBC patients. In this review, we identified that the histopathological manifestations of liver biopsy, the biochemical response to UDCA, the normalization of biomarkers like ALP, the efficacy of pruritus management, and the degree of cirrhosis as reflected by LSM are all the primary determinants for considering intensive therapy. Increasing studies are focusing on exploring the early determinants of PBC progression and gradually moving the timeline forward for risk stratification from 1 year to sooner, which enables individualized precision therapy, including disease surveillance and adjustment of treatment regimens, to be initiated as early as possible and integrated throughout the whole process (Fig. 1).

In conclusion, treatment for PBC should be pursued earlier, more precisely, more individualized optimal therapeutic schemes under higher stringent standards and with timely evaluations, aiming to enhance treatment efficacy and reduce the risk of unfavorable prognosis.

## Data Availability

No datasets were generated or analysed during the current study.
